# Supplementary material for: Reminiscent capillarity in subnanopores
Source: Nat Commun. 2019 Oct 11;10:4642. doi: 10.1038/s41467-019-12418-9 (PMC6789112; doi:10.1038/s41467-019-12418-9)
Supplement: Supplementary file 1 — Supplementary Information [file 41467_2019_12418_MOESM1_ESM.pdf]

Supplementary Information

Reminiscent Capillarity in Subnanopores

Deroche et al.

Supplementary Table 1. Physico-chemical properties of the investigated adsorbates. All data are reported at 298 K except for N_2 which are given at 77 K.

SOLVENT	Nitrogen	Acetone	p-xylene	n-hexane
Saturated vapor pressure @298K (except for N_2 @77) (kPa)	101.33	30.40	1.17	20.03
Molecular volume in bulk phase (nm^3 $molecule^{-1}$)	0.0576	0.1229	0.2045	0.2185
Surface tension ($mJ\ m^{-2}$)	9.0	25.2	27.76	18.43
Density ($mol\ dm^{-3}$)	28.78	13.64	8.12	7.62
Kinetic diameter - σ (nm)	0.36	0.46	0.59	0.43

Supplementary Table 2. Structural and textural parameters of the investigated zeosil structures. The reported values are extracted from the IZA database except the Henry coefficients for the nitrogen adsorption in the zeosil structures, estimated from the linear portions of the simulated adsorption isotherms within the low pressure domain (reported in Supplementary Figure 4).

ZEOSIL	BETAPA	CHA	MFI	STT
Pore openings (Å)	6.6 × 6.7<br>5.6 × 5.6	3.8 × 3.8	5.1 × 5.5<br>5.3 × 5.3	3.7 × 5.3<br>2.4 × 3.5
Framework density	1.57	1.56	1.83	1.69

(g cm^{-3})				
Surface Area (m^2 g^{-1})	874	686	439	447
Porous Volume (cm^3 g^{-1})	0.28	0.29	0.18	0.23
Accessible Volume (%)	20.5	17.3	9.8	13.9
K_H for N_2 @ 77K (mol kg^{-1} Pa^{-1})	1.4	95.6	605.7	72.8

Supplementary Table 3. Applied force field parameters for the zeosil and the adsorbate molecules.

Parameters for the non-bonded interactions			
Pseudo-atom	ε/k_B (K)	σ (Å)	q(e)
$CH_3(sp^3)$(n-hexane, acetone)	98	3.75	0
$CH_2(sp^3)$ (n-hexane)	46	3.95	0
$CH_3(sp^3)$ (p-xylene)	98	3.75	+0.095
CH(aro) (p-xylene)	50.5	3.695	0
(CH3-)C(aro) (p-xylene)	50.5	3.695	-0.095
O(=C) (acetone)	79	3.05	-0.424
C(=O) (acetone)	21	3.88	+0.424
N (nitrogen)	36	3.31	-0.482
com(N_2) (nitrogen)	0	0	+0.964
O (zeolite)	93.53	3.0	-1.2
Si (zeolite)	0	0	+2.4
Parameters for the bond potential			
Bond lengths (rigid bond)			

Type		r_0 (Å)		
CH_3-CH_2 (n-hexane)		1.54		
CH_2-CH_2 (n-hexane)		1.54		
O(=C)-C(=O) (acetone)			1.229	
CH_3-C(=O) (acetone)			1.52	
CH(aro)-CH(aro) (p-xylene)			1.4	
CH_3-C(aro) (p-xylene)			1.54	
N-com(N_2) (nitrogen)			0.55	
N-N (nitrogen)			1.1	
Bending potential parameters<br>$U_{bending} = k_\theta (\cos\theta - \cos\theta_{eq})$				
Type		θ_{eq}(°)	k_θ/k_B (K)	
CH_3-CH_2-CH_2 (n-hexane)		114.0	31250	
CH_3-C(=O)-CH_3 (acetone)		117.2	31250	
CH_3-C(=O)-O(=C) (acetone)		107.0	8800	
Rigid Angle				
Type		θ_{eq}(°)		
C(aro)-CH(aro)-CH(aro) (p-xylene)		120		
N-com(N_2)-N (nitrogen)		180		
Dihedral Torsion potential parameters (n-hexane)<br>$U_{torsion} = C_0 + C_1 (1+\cos\theta) + C_2 (1-\cos2\theta) + C_3 (1+\cos3\theta)$				
Type	C_0/k_B (K)	C_1/k_B (K)	C_2/k_B (K)	C_3/k_B (K)
CH_3-CH_2-CH_2-CH_2	0.0	355.03	-68.19	791.32
CH_2-CH_2-CH_2-CH_2	0.0	355.03	-68.19	791.32

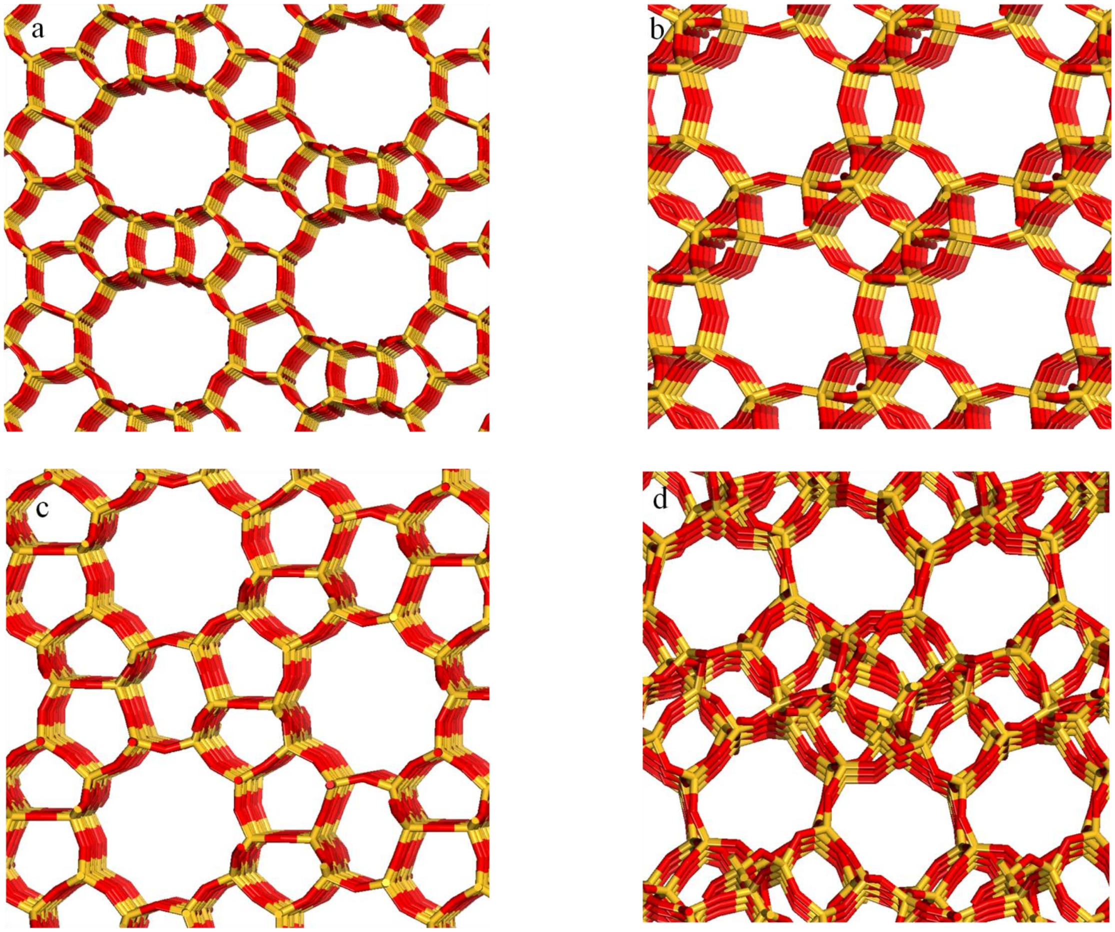

Supplementary Figure 1. Pore structure of the investigated purely siliceous zeolites. (a) Beta polymorph A (BETAPA) – viewed along the yz plan, (b) chabazite (CHA) – viewed along the xy plan, (c) silicalite-1 (MFI) viewed along the xz plan and (d) STT viewed along the xy plan. The yellow and red segments indicate the bonds between the O and Si atoms.

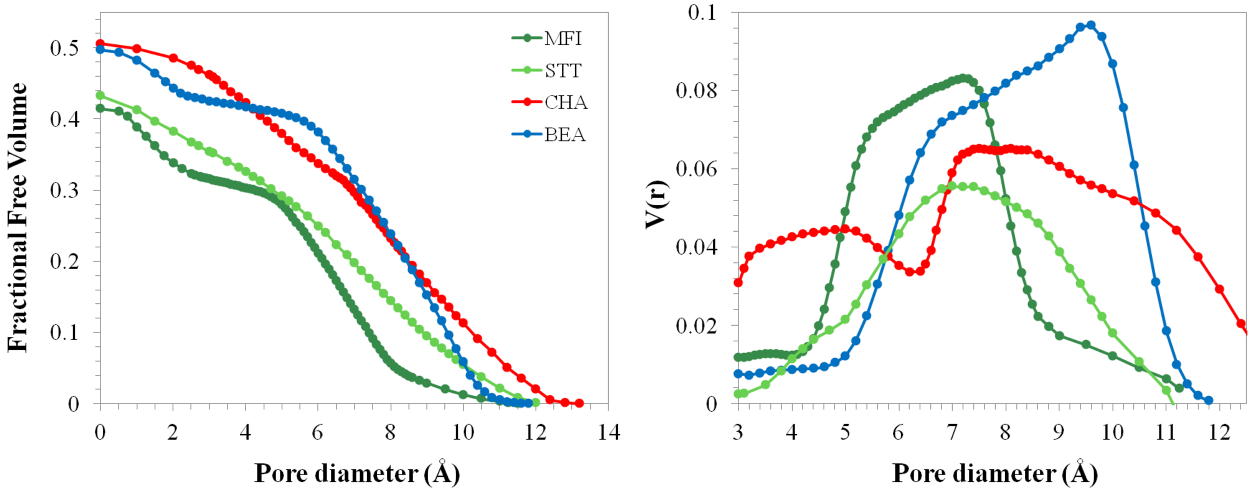

Supplementary Figure 2. **Estimation of the investigated zeosil pore size.** The evolution of the fractional free volume as a function of the diameter of the probe calculated using the Connolly surface area (left). The pore size distributions of the investigated zeolite structures derived from the fractional free volume curve (right). The following color code is used: dark green – silicalite-1 (MFI), red – chabazite (CHA), light green – STT and blue – beta polymorph A (BETAPA). The pore size distributions have been estimated as a derivative of the fractional free volume with respect to the diameter of the probe.

Supplementary Discussion

Supplementary Figure 3 compares the experimentally measured and simulated adsorption isotherms of nitrogen at 77 K within the 4 investigated zeosils, the adsorbed amounts have been normalized to the total number of adsorbed molecules N_0, corresponding to saturated porosity of each zeosil. Whereas the CHA, the *BEA and the STT exhibit an isotherm of type I [1], the experimental nitrogen adsorption isotherm within the silicalite-1 presents at the relative pressure of ~0.2 a characteristic step, typically explained by the adsorption induced transition from the initial orthorhombic lattice with the Pnma space group to another orthorhombic lattice with the $P2_12_12_1$ space group [2]. Thus, in order to reproduce the experiment, a composite simulated isotherm is plotted where the simulation of adsorption was achieved using the ORTHO framework (Pnma) in the lower pressure domain ($p/p_0 < 0.2$) and the PARA framework ($P2_12_12_1$) in the higher pressure domain ($p/p_0 > 0.2$).

First, we compare the experimentally measured maximum attained loadings (i.e. the plateau values) to the simulated ones. A very good agreement is observed between experiment and simulation for the Chabazite structure (~8.4 molecules per unit cell and ~8.3 molecules per unit cell obtained respectively from experiment and CBMC simulation) as well as for the silicalite-1 (~5.2 molecules per unit cell obtained by both experiment and simulation for the PARA framework). In contrast, we observe some discrepancy between the simulated and experimentally measured maximum loading for the STT (6.4 molecules per unit cell experimentally measured and 5.5 molecules per unit cell simulated) and for the *BEA-type zeosils (6.2 molecules per unit cell experimentally measured and 8 molecules per unit cell simulated). The difference between experimentally measured and simulated saturation loading

for the *BEA-type zeolite can be explained by the relatively low crystallinity (ranging around 80 %) of the *BEA-type sample, used to the experimentally measured adsorption isotherm [3]. Consequently, if we correct the experimental adsorbed amount to a purely siliceous *BEA-type zeolite with an ideal crystallinity, we obtain a saturation adsorption amount of 7.8 molecules per unit cell, which is perfectly coherent with the simulated result. Furthermore, since all the investigated zeolite structures have been described via a unique set of forcefield parameters without any adjustments, we consider that generally, the simulated results reasonably agree with the experiment. Whereas the plateau value corresponding to the saturation loading mostly depends on the adsorbate accessible pore volume (Supplementary Table 2), the initial part of the isotherm is related to the affinity of the adsorbate for the adsorbing surface. All the zeolites investigated in the present study are in their purely siliceous form; consequently their surfaces differ uniquely by the geometrical parameters. When zooming in the low pressure domain of the simulated isotherms (Supplementary Figure 3, top left inserted figure), we can roughly distinguish three different affinities: a high affinity for silicalite-1, an intermediate one for both STT and CHA and a low one for BETAPA. Such affinities are quantified through the Henry constant, extracted from the slope of the initial, linear portion of the adsorption isotherm. The values extracted from simulated isotherms are given in Supplementary Table 2 and the linear portions of those isotherms used for the extraction of Henry coefficients are reported in Supplementary Figure 4. Whereas the highest Henry constant value for the adsorption of nitrogen (dimensionless size, because extracted from the slope of the relative adsorbed quantity (N/N_0) as function of the relative pressure (p/p_0)) is obtained in silicalite-1 (K_H' = 10^7), followed by both CHA and STT (K_H' = 10^6), the lowest value was observed for *BEA-type zeosil (K_H' =$1.7{\times}10^4$). The evolution of isosteric heat of adsorption as a function of the adsorbed amount is

shown in the inserted figure on the right side of the Supplementary Figure 3. On the one hand we can observe a nearly constant evolution of the isosteric heat of nitrogen adsorption with the adsorbed amount in all the investigated purely siliceous zeolite structures. On the other hand, as expected from the Henry constants, the isosteric heat of nitrogen adsorption is the highest for silicalite-1 (17.7 kJ mol^{-1}), intermediate for both STT (16.1 kJ mol^{-1}) and CHA (16 kJ mol^{-1}) and the lowest for beta (12.4 kJ mol^{-1}) purely siliceous zeolites. Consequently, K_H' as well as the isosteric heats confirm the affinity of nitrogen molecules for the zeolite surfaces decreases in the following order: silicalite-1 >> CHA, STT >> *BEA. Pham et al. observed the same order of experimental values of isosteric heat of adsorption of nitrogen in their joined experimental – simulation study, carried out at 303 K. However, these authors obtained slightly (about less than 10%) lower values (silicalite-1 – 16.2 kJ mol^{-1}; STT – 14.1 kJ mol^{-1}, CHA – 13.6 kJ mol^{-1} and *BEA - 11.2 kJ mol^{-1}) [4]. Similar isosteric heats were obtained by Fischer et al. in their simulation study of nitrogen adsorption in a series of zeosils [5] (silicalite-1 ~17 kJ mol^{-1}, CHA ~14.5 kJ mol^{-1} and BETAPA ~12.5kJ mol^{-1}). Furthermore, Grey et al. extracted through the GCMC simulation, performed at 298 K the zero coverage heat of adsorption of 17.5 kJ mol^{-1} (Kiselev model) and ~14.6 kJ mol^{-1} (PN model) for nitrogen adsorption in CHA [6], while Newsome et al. applying the same technique observed an isosteric heat of 14 kJ mol^{-1} for nitrogen in silicalite-1 at 308 K, remaining constant as a function of loading [7]. Li et al. determined through experimental gas chromatography measurement an isosteric heat of adsorption of 11.4 kJ mol^{-1} for the adsorption of nitrogen in *BEA-type zeolite [8]. Generally, our results compare well with the already published values; the differences remain below 10%. Considering the structural parameters reported in Supplementary Table 2, one can notice the affinity of the nitrogen molecule for the investigated zeolite surfaces roughly follows the inverse

order of the accessible volume ratio. Lowering accessible volume ratio implies increasing framework ratio (and thus decreasing porosity) in consequence the interaction adsorbate / adsorbent becomes stronger. Furthermore, the nitrogen affinity does not seem to be influenced by the zeolite pore size distribution (Supplementary Figure 2) that could be explained by relatively small dimensions of the molecule compared to the considered zeolites pore openings.

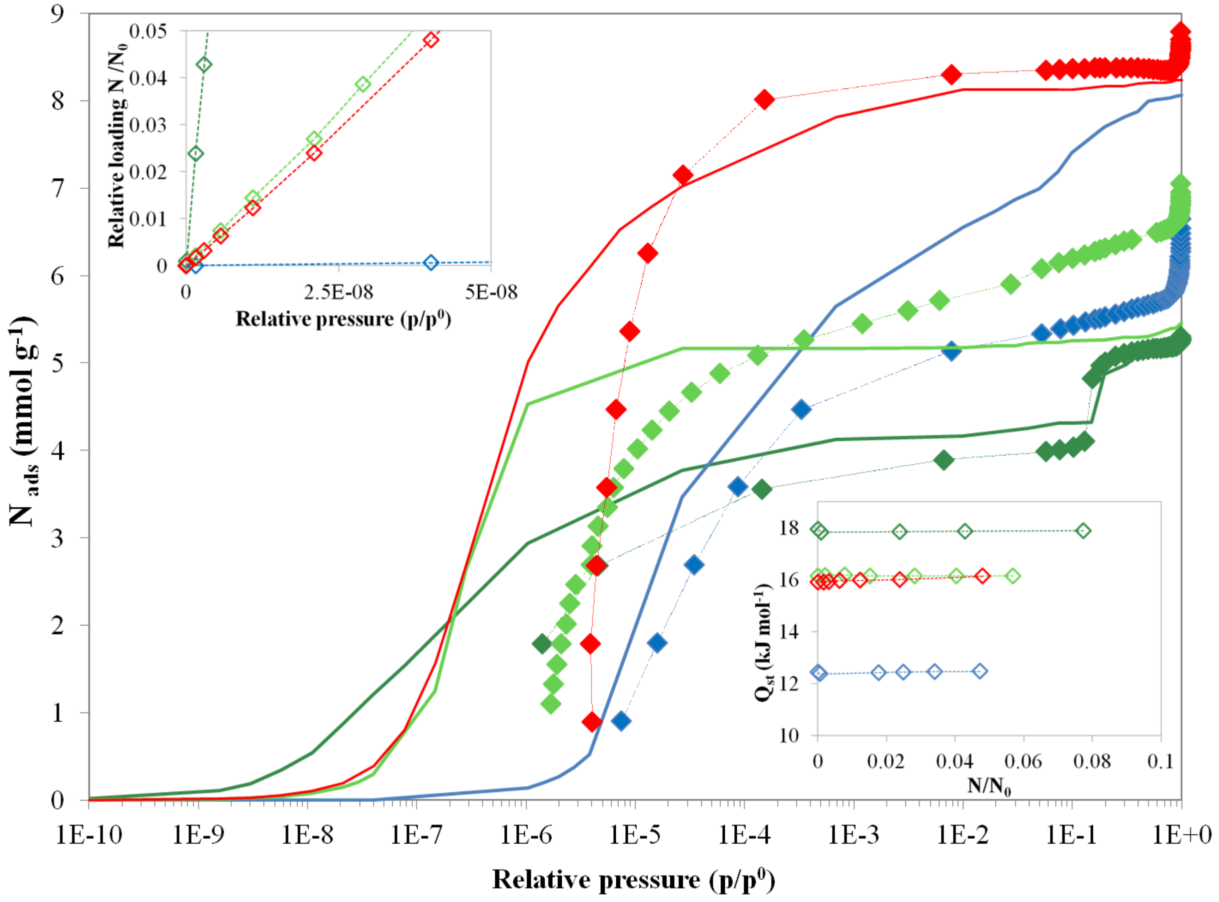

Supplementary Figure 3. Experimental and simulated nitrogen adsorption isotherms in zeosils at 77 K. The lines correspond to simulated data while the full symbols correspond to the experimental values. The color code is dark green – silicalite-1 (MFI), red – chabazite (CHA), green – STT and blue – beta (*BEA). The top left inserted figure presents the low pressure domain simulated isotherms. The lower right inserted figure shows the evolution of the isosteric heats of adsorption simulated as a function of the adsorbed amount.

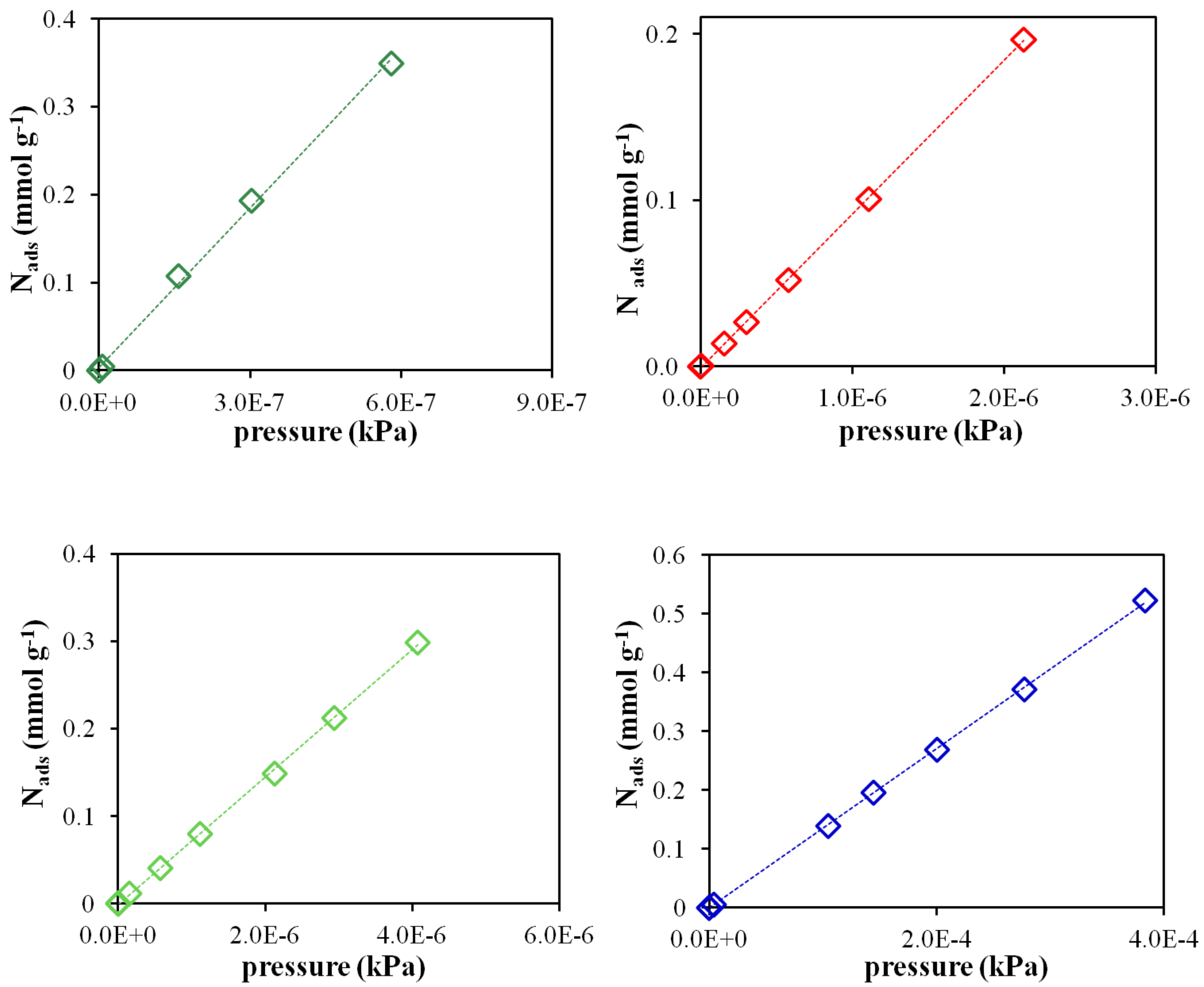

Supplementary Figure 4. The low pressure domain isotherms of nitrogen. Isotherms are reported for following zeosil structures: (top-left) silicalite-1 (MFI), (top right) chabazite (CHA), (bottom left) STT and (bottom right) beta (BETAPA).

Figure 3 shows the simulated adsorption isotherms for acetone, n-hexane and p-xylene in the 4 investigated zeolites at room temperature (298 K). We also show the Henry constants extracted from the initial, linear portions of isotherms are reported in the Supplementary Table 4.

Several adsorbate-adsorbent couples under study have already been investigated by various experimental and/or simulation techniques thus we afforded a comparison with the already published results. Jakob et al. measured experimentally the total adsorption capacity of 1.39 and 1.19 mmol g^{-1} respectively for n-hexane and p-xylene in the purely siliceous *BEA-type zeolite at 298 K [9]. A slightly lower porous volume of their investigated zeolite sample (nitrogen adsorption capacity at 77 K of 7.14 mmol g^{-1} achieved experimentally against 8.0 mmol g^{-1} from our simulation) could explain the difference between their experimental and our simulated solvent adsorption capacities. If we correct our simulated adsorbed loadings by the experimental/simulated porous volumes ratio, we obtain a loading 1.4 mmol g^{-1} for the n-hexane and p-xylene adsorption in the BETAPA-type zeolite. Further, for the adsorption of p-xylene in silicalite-1 from the experimental point of view, the total adsorption capacity of ~1.4 mmol g^{-1} was observed from thermogravimetry by Lee et al. at 303 K [10] and Ban et al. at 323 K [11]. For the adsorption of n-hexane in silicalite-1 Song et al. obtained the saturation capacity of 8 molecules per unit cell (~1.39 mmol g^{-1}) [12,13], and Krishna observed the same result applying the CBMC simulation at 298 K [14]. Consequently, our total adsorbed amount is in perfect agreement with previously published results.

Supplementary Figure 5 reports the evolution of the isosteric heat of adsorption as a function of loading and the values of isosteric heats extrapolated to zero loading are summarized in Supplementary Table 5. We have compared our values of isosteric heats to the literature ones. Song et al. [12] found from simulation for the adsorption of p-xylene in silicalite-1 at 673 K the isosteric heats of 76.8 and 69.1 kJ mol^{-1} applying respectively the Buchart-Dreiding and PCFF force-fields. Song obtained experimentally the isosteric heat of ~64 kJ mol^{-1} for the n-hexane adsorption in silicalite [13]. Sepa et al. measured experimentally by microcalorimetry the

differential heat of adsorption of acetone in silicalite at 350 K of 67 ± 3 kJ mol^{-1} [15]. Thus, the already published values for heat of adsorption compare well with our simulated values. A detailed inspection of the curve of evolution of the isosteric heat of adsorption with loading shows a slightly increasing trend, usually observed in VOCs adsorption in purely siliceous zeolites [14].

Supplementary Table 4 Simulated Henry Constants (K_H' in mol kg^{-1}) for adsorption of n-hexane, acetone and p-xylene in investigated zeosils. The values have been obtained by fitting the low pressure linear parts of the adsorption isotherms simulated at the room temperature (298 K), expressed as the adsorbed quantity in function of the relative pressure (p/p_0). The values of saturated vapor pressures (p_0) at 298 K for investigated volatile organic compound are listed in Supplementary Table 1.

VOC	MFI	CHA	STT	BETAPA
acetone	3.3×10^4	1.8×10^4	1.0×10^4	0.2×10^4
n-hexane	12.2×10^4	3.1×10^4	5.4×10^4	1.6×10^4
p-xylene	0.2×10^4	0.1×10^4	1.2×10^4	2.7×10^4

Supplementary Table 5 Extrapolated "zero loading" isosteric heat of adsorption (kJ mol^{-1}) for acetone, n-hexane and p-xylene adsorption in investigated zeosils.

VOC	MFI	CHA	STT	BETAPA
n-hexane	59.1	46.2	49.5	43.2
acetone	63.1	55.1	55.8	44.8
p-xylene	72.8	67.3	69.5	65.8

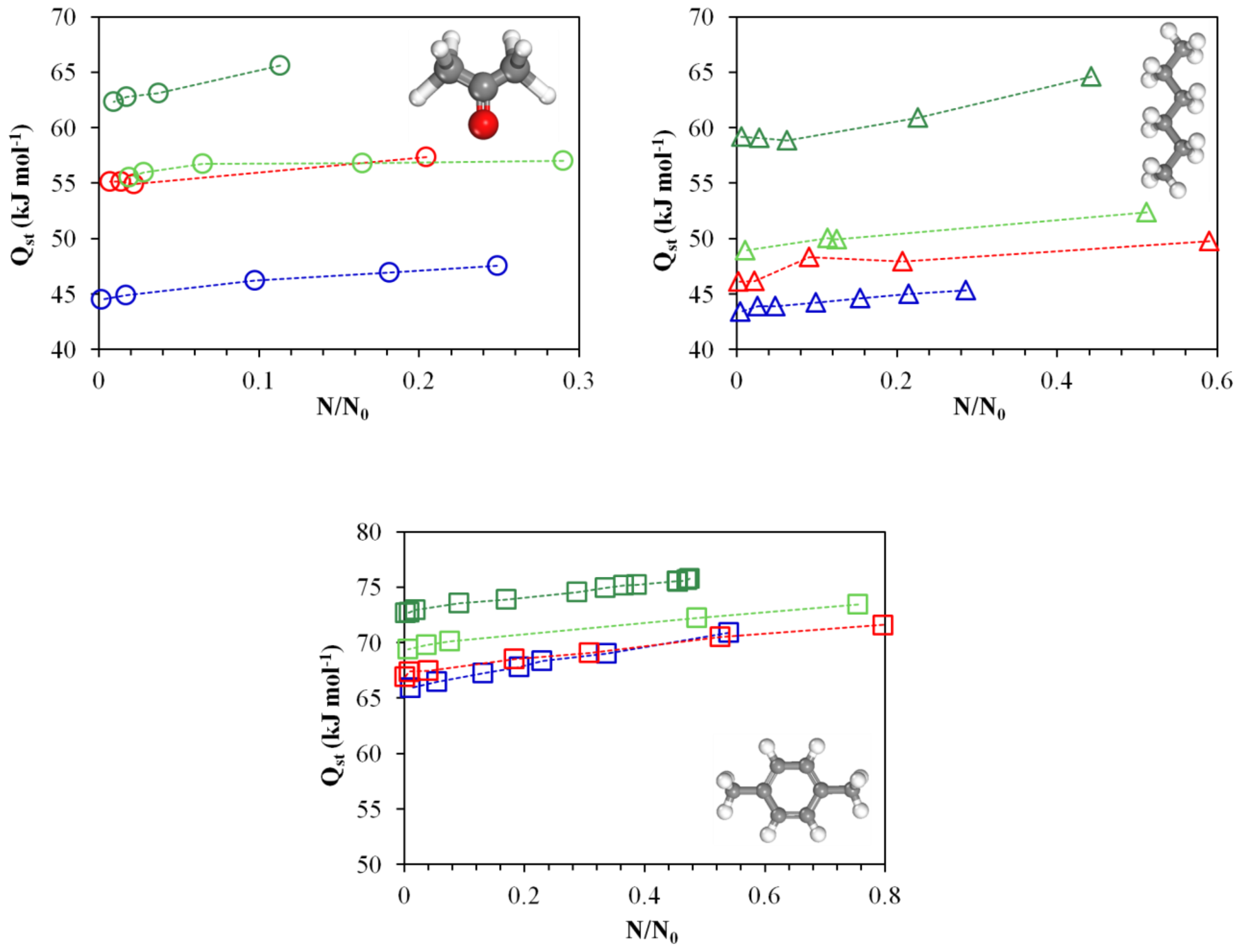

Supplementary Figure 5. Isosteric heat of adsorption as function of the loading. Data are reported for acetone (top left), n-hexane (top right) and p-xylene (in the bottom) adsorbed in purely sileceous zeosils with following color code: dark green - silicalite (MFI), red - chabazite (CHA), light green- STT and blue - BETAPA. The adsorbed amounts have been normalized to the total number of adsorbed molecules N_0, corresponding to saturated porosity of each zeosil.

Moreover, we aim at rationalizing the total adsorption capacity of each zeolite for a given adsorbent. To that purpose we have reported in Supplementary Figure 6 the maximum adsorbed amount (i.e. the plateau value) as a function of the ratio of porous volume (V_{porous}) of the zeolite structure to the molecular volume ($V_{molecular}$) of given solvent. With the aim to compare the

zeolite structures on identical basis, the total adsorbed amount was expressed as a number of molecules per single SiO_2 unit. One can observe a linear evolution of the total adsorbed amount as a function of the ratio $V_{porous}/V_{molecular}$, relation known as Gurvich's rule [11].

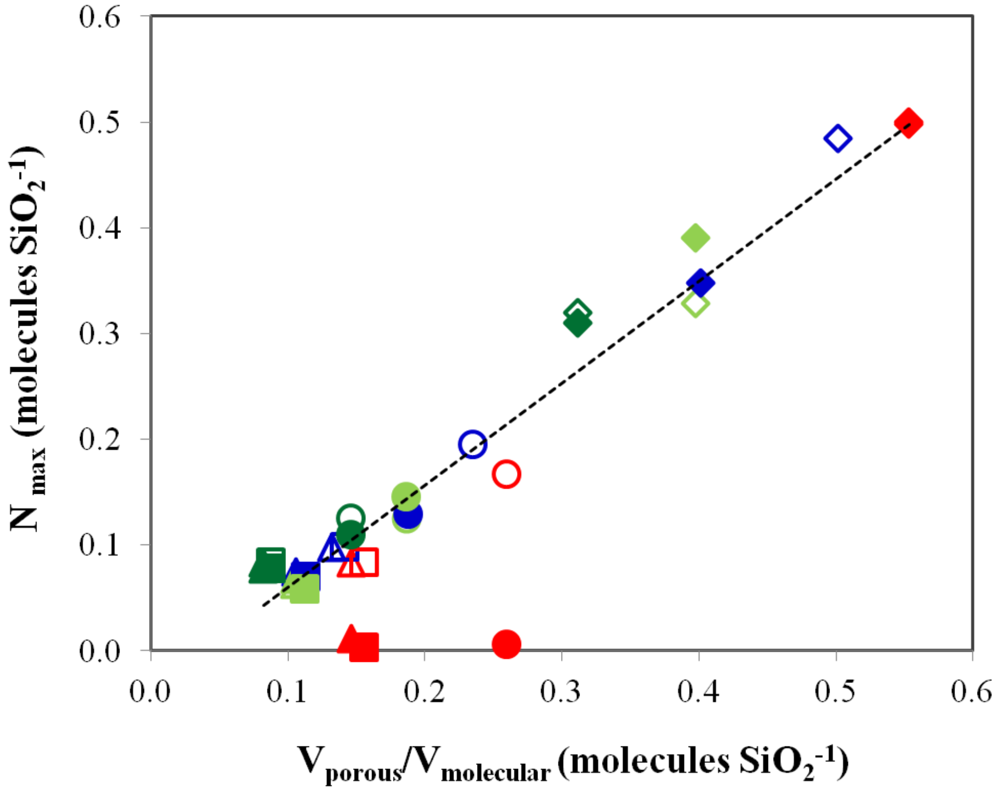

Supplementary Figure 6. Total adsorbed amount as a function of the ratio $V_{porous}/V_{molecular}$. The maximum adsorbed amount is reported as the number of adsorbed molecules per single SiO_2 unit for: nitrogen - diamond, acetone - circle, p-xylene - triangle and n-hexane - square in 4 siliceous zeolites: MFI - dark green, BETAPA - blue, STT - light green and CHA - red as a function of the ratio $V_{porous}/V_{molecular}$. Filled symbols correspond to our experimental data while empty symbols represent our CB-GCMC simulation data. The experimental points for *BEA were calculated with the experimentally measured porous volume which was 20% lower than the theoretical one because of its imperfect crystallinity [13]. The discrepancy between

simulated/experimental data for COV (p-xylene, acetone, n-hexane) in CHA can be explained by the chabazite narrow channels structure whose size is comparable to the kinetic diameter of the adsorbates. For this sample, our experiments demonstrate that the adsorption kinetics is extremely slow so that they fail to reach equilibrium (the measured adsorbed amount increases upon increasing the temperature – which is impossible from a thermodynamic viewpoint).

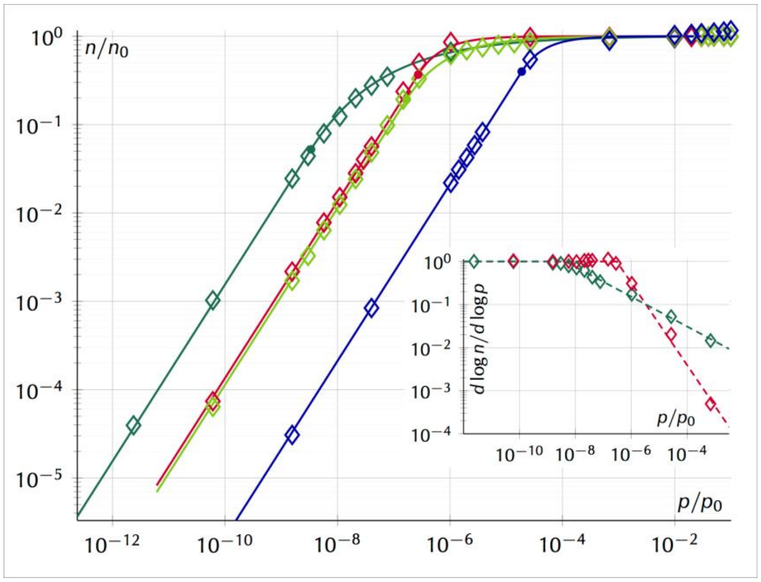

Supplementary Figure 7. Adsorption isotherms for nitrogen in log log scale. The relative adsorbed quantity (n/n_0) is expressed in function of the relative pressure (p/p_0), where p_0 corresponds to the saturation pressure. The isotherms reveal a two regime behavior: a linear Henry's type regime at low pressure followed by an exponential dependence of log n according to the pressure as shown from the slope according to p/p_0 plotted in the inset in a log log scale. Dots are simulated points; lines are fits of a Henry/exponential behavior.

Writing $\eta = \ln(n/n_0)$ as the logarithm of the dimensionless fluid quantity in the pore and $x = \ln(p/p_0)$ the logarithm of the relative pressure so that $-\infty < \eta < 0$ and $-\infty < x < 0$. The behavior observed in log log plot corresponds to :

$$\eta = x + b \qquad\qquad x < x_f$$

$$\eta = \frac{1-e^{-kx}}{ke^{-kx_f}} \qquad\qquad x \geq x_f$$

With b the logarithm of the coefficient of the Henry type law that characterized the adsorption at low pressure such that $x < x_f$ with x_f associated to the pressure p_f at which the filling of the pore begins. The exponential behavior observed for $x > x_f$ is such that the logarithm of the slope $\ln(\mathrm{d}\eta/\mathrm{d}x) = -k\,(x - x_f)$ varies linearly with x with a slope k and is equal to 0 for $x = x_f$ as shown in the inset of the figure 5 in the paper. This ensures the continuity of the derivative such that $\mathrm{d}\eta/\mathrm{d}x = 1$ for $x < x_f$ and $x = x_f$. The continuity of η for $x = x_f$ imposes as well:

$$x_f + b = \frac{1 - e^{-kx_f}}{ke^{-kx_f}} = \frac{1}{k}\,(e^{kx_f} - 1)$$

To find the numerical value of x_f the previous relation is transformed as:

$$k\,(x_f + b)\,e^{-kx_f} = 1 - e^{-kx_f}$$

which is equivalent to

$$(k\,(x_f + b) + 1)\,e^{-kx_f} = 1$$

Or

$$-(k\,(x_f + b) + 1)\,e^{-(k(x_f+b)+1)} = -e^{-(kb+1)}$$

The value of x_f is found from the value of $z = -\,(k\,(x_f + b) + 1)$, using the Lambert W function defined such that $z = W(ze^z)$, that is:

$$x_f = -\frac{1}{k}\left(1 + W\left(-e^{-kb+1)}\right)\right) - b \qquad (1)$$

The value of x_f is found from Supplementary Equation 1 knowing the values of k and b. The value of b is identified as the logarithim of the slope of n/n_0 according to p/p_0 at low pressure and the value of k is identified as the slope of ln (dη/dx) according to x at high pressure.

Supplementary References

[1] Rouquerol, J., Rouquerol, F., Llewellyn, P., Maurin, G., Sing, K.S.W. Adsorption by Powder and Porous Solids (Second Edition) Principles, Methodology and Applications, Academic Press, Oxford (UK) 2014.

[2] Nicholson, D.; Pellenq, R. Adsorption in zeolites: intermolecular interactions and computer simulation. *Adv. Colloid Interface Sci.* **76-77**, 179-202 (1998).

[3] Cosseron, A.F.; Daou, T. J. ; Tzanis, L.; Nouali, H.; Deroche, I. ; Coasne, B.; Tchamber, V. Adsorption of volatile organic compounds in pure silica CHA, *BEA, MFI and STT-type zeolites. *Microporous Mesoporous Mater.* **173**,147-154 (2013).

[4] Pham, T.D.; Xiong, R.X.; Sandler, S.I.; Lobo, R.F. Experimental and computational studies on the adsorption of CO2 and N2 on pure silica zeolites. *Microporous Mesoporous Mater.* **185**, 157-166 (2014).

[5] Fischer, M.; Bell, R. G. Influence of Zeolite Topology on CO2/N2 Separation Behaviour: Force-Field Simulations Using a DFT-Derived Charge Model. *J. Phys. Chem. C* **116**, 26449-26463 (2012).

[6] Grey, T.J.; Travis, K.P.; Gale, J.D., Nicholson, D. A comparative study of the adsorption of nitrogen and methane in siliceous heulandites and chabazite. *Microporous Mesoporous Mater.* **48**, 203-209 (2009).

[7] Newsome, D.; Gunawan, S.; Baron, G.; Denayer, J.; Coppens, M.-O. Adsorption of CO_2 and N_2 in Na-ZSM-5: effects of Na^+ and Al content studied by Grand Canonical Monte Carlo simulations and experiments. *Adsorption* **20**, 157-171 (2014).

[8] Li, P.; Tezel, H. Adsorption separation of N_2, O_2, CO_2 and CH_4 gases by beta-zeolite. *Microporous Mesoporous Mater.***98**, 94-101 (2007).

[9] Jakob, A.; Valtchev, V.; Soulard, M.; Faye, D. Synthesis of Zeolite Beta Films in Fluoride Media and Investigation of Their Sorption Properties. *Langmuir* **25**, 3549-3555 (2009).

[10] Lee, C-K.; Chiang, A.S.T. Adsorption of aromatic compounds in large MFI zeolite crystals. *J. Chem. Soc., Faraday Trans.* **92**, 3445-3451 (1996).

[11] Ban, H.; Gui, J.; Duan, L.; Zhang, X.; Song, L.; Sun, Z. Sorption of hydrocarbons in silicalite-1 studied by intelligent gravimetry. *Fluid Phase Equilib.* **232**, 149-158 (2005).

[12] Song, L.; Sun, L.-Z.; Rees, L.V.C. Experimental and molecular simulation studies of adsorption and diffusion of cyclic hydrocarbons in silicalite-1. *Microporous Mesoporous Mater.* **55**, 31-49 (2002).

[13] Song, L.; Sun, Z.; Duan, L.; Gui, J.; McDougall, G.S. Adsorption and diffusion properties of hydrocarbon in zeolites. *Microporous Mesoporous Mater.* **104**, 115-128 (2007).

[14] Krishna, R. Evaluation of Procedures for estimation of the isosteric heat of adsorption in microporous materials. *Chem. Eng. Sci.* **123**, 191-195 (2015).

[15] Sepa, J.; Lee, C.; Gorte, R.J.; White, D.; Kassab, E.; Evleth, E.M.; Jessri, H. and Allavena, M. Carbonyl ^{13}C Shielding Tensors and Heats of Adsorption of Acetone Adsorbed in Silicalite and the 1:1 Stoichiometric Complex in H-ZSM-5. *J. Phys. Chem.* **100**, 18515-18523 (1996).
