# Supplementary material for: Reminiscent capillarity in subnanopores
Source: Nat Commun. 2019 Oct 11;10:4642. doi: 10.1038/s41467-019-12418-9 (PMC6789112; doi:10.1038/s41467-019-12418-9)
Supplement: Supplementary file 3 — Description of Additional Supplementary Files [file 41467_2019_12418_MOESM3_ESM.pdf]

Description of Additional Supplementary Files

File Name: Supplementary Data 1
Description: Structure of the BEA zeolite in .car format.

File Name: Supplementary Data 2
Description: Structure of the CHA zeolite in .car format.

File Name: Supplementary Data 3
Description: Structure of the MFI zeolite in .car format.

File Name: Supplementary Data 4
Description: Structure of the STT zeolite in .car format.
